# Supplementary material for: The satiety hormone cholecystokinin gates reproduction in fish by controlling gonadotropin secretion
Source: eLife. 2024 Dec 24;13:RP96344. doi: 10.7554/eLife.96344 (PMC11668526; doi:10.7554/eLife.96344)
Supplement: Figure 1—source data 1. [file elife-96344-fig1-data1.docx]

| **Guide #** | **Sequence (5’ 🡪 3’)** | **Primers for HRM screening**  **Sequence (5’ 🡪 3’)** |
| --- | --- | --- |
| 1 | ACAACGCGTCGATTCTGAAC | \| Forward: AACAGTTGTGGTCAGGTCCG \| \| --- \| \| Revers: CGCATTTGACAGAAAGCGCA \| |
| 2 | TAGGATACGGCGTCATAATG | \| Forward: TGCTTTGCGAGTGAAATGCTGA \| \| --- \| \| Revers: GCCTTCTCCACCTCGCTATTCT \| |
| 3 | CCCAACAGGCTCCGCAAAGG | \| Forward: TCTTCTGCTACCTCACAAGCACT \| \| --- \| \| Revers: CGGATCACTCGCTTCTTGGC \| |
| **Primers for real time PCR of tissue distributions, sequence (5’ 🡪 3’)** | | |
| TiCCKAR-F | CAAGGTCATCACTGCCACCT |  |
| TiCCKAR-R | GGGACACATACCAGGACTGC |  |
| TiCCKBR-F | GTCACACTCTGCCTGGTCTC |  |
| TiCCKBR-R | CATAACAGCCGGAGAAGGGG |  |
| TiCCKBRA-F | ACATCCATCAACCCCGAGTG |  |
| TiCCKBRA-R | GAGCAGGATCCGAAGTGTGT |  |
