# Supplementary material for: The satiety hormone cholecystokinin gates reproduction in fish by controlling gonadotropin secretion
Source: eLife. 2024 Dec 24;13:RP96344. doi: 10.7554/eLife.96344 (PMC11668526; doi:10.7554/eLife.96344)
Supplement: Figure 5—source data 1. [file elife-96344-fig5-data1.docx]

| **Gene** | **Sequence** | **Slope** | **R^2** |
| --- | --- | --- | --- |
| zfEF1A 954F | CTAGCCGTCCCACCGACAAG | -3.2 | 0.99 |
| zfEF1A 1151R | GCAGGCGATGTGAGCAGTGT |  |  |
| zfLHβ 190F | AATGCCTGGTGTTTCAGACC | -3.2 | 1 |
| zfLHβ 353R | AACAGTCGGGCACGTTAATG |  |  |
| zfFSHβ 190F | TGTGGGAGCTGCGTCACAAT | -3.2 | 0.99 |
| zfFSHβ 327R | GCCACGGGGTACACGGAAGAC |  |  |
